# A review and phylogeny of Scarabaeine dung beetle fossils (Coleoptera: Scarabaeidae: Scarabaeinae), with the description of two *Canthochilum* species from Dominican amber

**DOI:** 10.7717/peerj.1988

**Published:** 2016-05-11

**Authors:** Sergei Tarasov, Fernando Z. Vaz-de-Mello, Frank-Thorsten Krell, Dimitar Dimitrov

**Affiliations:** 1 Department of Research and Collections, Natural History Museum, University of Oslo, Oslo, Norway; 2 Departamento de Biologia e Zoologia, Instituto de Biociências, Universidade Federal de Mato Grosso, Cuiabá, Mato Grosso, Brazil; 3 Department of Zoology, Denver Museum of Nature & Science, Denver, Colorado, USA

**Keywords:** Dung beetles, Scarabaeinae, *Canthochilum*, New species, Fossils, Phylogeny, Catalogue, Coleoptera

## Abstract

Despite the increasing rate of systematic research on scarabaeine dung beetles (Coleoptera: Scarabaeidae: Scarabaeinae), their fossil record has remained largely unrevised. In this paper, we review all 33 named scarabaeine fossils and describe two new species from Dominican amber (*Canthochilum alleni* **sp.n.**, *Canthochilum philipsivieorum* **sp.n.**). We provide a catalogue of all fossil Scarabaeinae and evaluate their assignment to this subfamily, based primarily on the original descriptions but also, where possible, by examining the type specimens. We suggest that only 21 fossil taxa can be reliably assigned to the Scarabaeinae, while the remaining 14 should be treated as doubtful Scarabaeinae. The doubtful scarabaeines include the two oldest dung beetle fossils known from the Cretaceous and we suggest excluding them from any assessments of the minimum age of scarabaeine dung beetles. The earliest reliably described scarabaeine fossil appears to be *Lobateuchus parisii*, known from Oise amber (France), which shifts the minimum age of the Scarabaeinae to the Eocene (53 Ma). We scored the best-preserved fossils, namely *Lobateuchus* and the two *Canthochilum* species described herein, into the character matrix used in a recent morphology-based study of dung beetles, and then inferred their phylogenetic relationships with Bayesian and parsimony methods. All analyses yielded consistent phylogenies where the two fossil *Canthochilum* are placed in a clade with the extant species of *Canthochilum*, and *Lobateuchus* is recovered in a clade with the extant genera *Ateuchus* and *Aphengium*. Additionally, we evaluated the distribution of dung beetle fossils in the light of current global dung beetle phylogenetic hypotheses, geological time and biogeography. The presence of only extant genera in the late Oligocene and all later records suggests that the main present-day dung beetle lineages had already been established by the late Oligocene–mid Miocene.

## Introduction

Scarabaeine dung beetles (Coleoptera: Scarabaeidae: Scarabaeinae) are a primarily dung-feeding subfamily comprising ∼6,200 species and ∼267 genera worldwide (Tarasov & Génier, 2015). Recently, this subfamily was the subject of 15 key studies using molecular and morphological data (summarized in Scholtz (2009b) and Tarasov & Génier (2015)) that aimed at constructing a robust phylogeny and/or at facilitating comparative evolutionary studies in dung beetles. Further development of scarabaeine systematics and evolutionary research would benefit greatly from analytical approaches that integrate fossils with morphology and molecules for combined phylogenetic inference (Ronquist et al., 2012b; Warnock et al., 2015). While our knowledge of molecular and morphological evolution of dung beetles has grown considerably over the last decade, their fossil record has remained almost unstudied. Nevertheless, fossils represent an essential data source for resolving relationships, understanding morphological character evolution and assessing the tempo and mode of diversification. Ignoring the fossil record makes tree calibration procedures less robust methodologically (Ronquist et al., 2012a; Ronquist et al., 2012b). Currently, inference of a time-annotated evolutionary history of dung beetles is hampered by the lack of a detailed investigation of their known fossils. In the present study we aim to address this issue by providing a critical overview of the fossil record of the group.

Fossil Scarabaeinae are rare in collections, with just 33 described species prior to this study. Here, we describe two additional species of the genus *Canthochilum* from Dominican amber (16 Ma), bringing the total number of fossil species to 35. Such a scarce fossil record is likely the result of taphonomic biases, rather than a lack of paleontological studies on this group. Herein, we review the scarabaeine fossil record and provide a catalogue of all described species, which is updated from previous works (Krell, 2000a; Krell, 2007) and supplemented with notes on the credibility of the fossils’ taxonomic placement. Due to the poor preservation of fossilized specimens important characters are often missing, which often leads to misidentifications. In this paper, we examine the original descriptions and illustrations of all described scarabaeine fossil species and evaluate the potential of misidentifications in the original taxonomic placements. Because proper taxonomic placement needs an investigation of type specimens and we could not obtain the types of all 33 fossil taxa known prior to this study, the notes on potential issues with taxa for which the types were not examined do not represent formal taxonomic decisions. However, they raise flags where caution and further taxonomic work is necessary. The preserved or documented characters of 14 of the fossils currently described as scarabaeine do not allow their unambiguous placement in Scarabaeinae. Available evidence does support the placement of the remaining 21 fossils species (including the two new species described herein) in Scarabaeinae. We discuss the distribution of these 21 species across the scarabaeine phylogeny and through geological time. While most reliable fossil scarabaeines belong to extant genera, one of them, *Lobateuchus parisii* from the Eocene (53 Ma), is a member of an extinct genus and is also the oldest reliably identified scarabaeine fossil. We argue that the minimal age for the Scarabaeinae should be aligned with the age of *Lobateuchus* and not with the ages of the oldest recorded doubtful “scarabaeines” known from the Cretaceous (i.e., *Prinocephale* Lin, 1980 and *Cretonitis* Nikolajev, 2007).

We selected the best-preserved dung beetle fossils, the *Canthochilum* species described herein and the oldest scarabaeine fossil *Lobateuchus,* all of which are known from amber, and added them to the most recent morphological character matrix of Scarabaeinae (Tarasov & Génier, 2015). Then, we analysed the resulting dataset (including both fossil and extant species) using parsimony and Bayesian methods of phylogenetic inference.

## Materials and Methods

### 

#### Examination of fossils

The Dominican amber pieces containing the fossilized remains of the two new species described herein were examined dry using a Leica MZ16 stereomicroscope. Photos were taken with a Canon EOS 500D digital camera attached to a Leica MZ16 stereomicroscope and with a Canon EOS 1DS Mark III attached to an Infinity K2/SC system. Several fossils described by Heer (1862) were studied from detailed photos kindly provided by the curators of the Geological-Paleontological Collection at Eidgenössische Technische Hochschule Zürich in Switzerland. The remaining non-amber fossils were examined using only literature sources, i.e. original descriptions, other relevant works and illustrations when available.

#### Deposition of fossils

The amber pieces that hold the described *Canthochilum* fossils are deposited in CEMT–Setor de Entomologia da Coleção Zoológica, Instituto de Biociências, Universidade Federal de Mato Grosso, Cuiabá, Brazil (curator of scarab collection Fernando Vaz-de-Mello). Another examined amber fossil, *Lobateuchus parisii,* is deposited at Muséum national d’Histoire naturelle, Paris (MNHN, curator of insect fossil collection André Nel).

#### Dating of fossils

The age of the fossils is derived from the latest publications discussing in depth the relevant deposits (Table 1). If applicable, absolute ages were adjusted to the current dating of epochs or stages as summarized by Cohen, Finney & Gibbard (2015) and Gradstein et al. (2012).

**Table 1:** List of the described fossil Scarabaeinae and the confidence of their placement in Scarabaeinae. The table puts the scarabaeine fossils in two categories–those which can be confidently assigned to Scarabaeinae and those whose assignment is doubtful. The column *Stat.* (Status) summarizes our confidence for treating a fossil as a member of Scarabaeinae and classifies them in the following categories: *(S)* true scarabaeine with correct generic placement; *(?S)* true scarabaeine but its generic placement needs a further investigation; *(DS)* doubtful scarabaeine, the description lacks any evidence for assigning of the fossil to Scarabaeinae; *(L)* the fossil specimen is presumed lost. The justification for placing a fossil in any of these four categories is given in the Catalogue section. The column *Age* provides the fossil age data (derived from the age of the strata or amber that contain the fossils) for those fossils that can be confidently placed in Scarabaeinae. The age information was retrieved from references listed in the *Dating source* column.

		Epoch	Age (Ma)	Dating source	Locality	Stat.
	**Fossils confidently assigned to Scarabaeinae**					
1	*Anachalcos mfwangani* Paulian, 1976	L–M Miocene	22–15	Drake et al. (1988) and Peppe et al. (2009)	Lake Victoria, Kenya	?S
2	*Canthochilum alleni* **sp.n.**	L Miocene	16	Iturralde-Vinent (2001)	Dominican amber	S
3	*Canthochilum philipsivieorum* **sp.n.**	L Miocene	16	Iturralde-Vinent (2001)	Dominican amber	S
4	*Copris kartlinus* Kabakov, 1988	U Miocene–L Pliocene	9.8–3.6	Aslanyan et al. (1982) and Adamia et al. (2010)	Kisatibi/Goderdzi/Kura formation, Georgia	S
5	*Copris druidum* Heer, 1862	M Miocene	14–13.5	Berger et al. (2005) and Kälin et al. (2001)	Öhningen, Germany	?S
6	*Copris leakeyorum* Paulian, 1976	L–M Miocene	22–15	Drake et al. (1988) and Peppe et al. (2009)	Lake Victoria, Kenya	?S
7	*Copris pristinus* Pierce, 1946	U Pleistocene	0.068–0.004 (pits: 0.068–0.008)	O’Keefe et al. (2009)	La Brea tar pits, U.S.A.	S
8	*Eodrepanus coopei* Barbero, Palestrini & Roggero, 2009	M–U Pleistocene	0.130–0.115	Preece (1999) and Dahl-Jensen et al. (2013)	Trafalgar Square, UK	S
9	*Gymnopleurus rotundatus* Heer, 1862	M Miocene	14–13.5	Berger et al. (2005) and Kälin et al. (2001)	Öhningen, Germany	?S
10	*Gymnopleurus sisyphus* Heer, 1847	M Miocene	14–13.5	Berger et al. (2005) and Kälin et al. (2001)	Öhningen, Germany	S
11	*Heliocopris antiquus* Fujiyama, 1968	L Miocene	23.03–18.7	Suzuki & Terada (1996)	Noto, Japan	S
12	*Lobateuchus parisii* Montreuil, Génier & Nel, 2010	L Eocene	53	Nel & Brasero (2010)	Oise amber, France	S
13	*Metacatharsius rusingae* Paulian, 1976	L–M Miocene	22–15	Drake et al. (1988) and Peppe et al. (2009)	Lake Victoria, Kenya	?S
14	*Onthophagus bisontinus* Heer, 1862	M Miocene	14–13.5	Berger et al. (2005) and Kälin et al. (2001)	Öhningen, Germany	S
15	*Onthophagus crassus* Heer, 1862	M Miocene	14–13.5	Berger et al. (2005) and Kälin et al. (2001)	Öhningen, Germany	?S
16	*Onthophagus everestae* Pierce, 1946	U Pleistocene	0.068–0.004 (pit: 0.030–0.009)	O’Keefe et al. (2009)	La Brea tar pits, U.S.A.	S
17	*Onthophagus ovatulus* Heer, 1847	M Miocene	14–13.5	Berger et al. (2005) and Kälin et al. (2001)	Öhningen, Germany	?S
18	*Onthophagus prodromus* Heer, 1862	M Miocene	14–13.5	Berger et al. (2005) and Kälin et al. (2001)	Öhningen, Germany	?S
19	*Onthophagus statzi* Krell, 1990	U Oligocene	25	Koenigswald et al. (1996)	Rott, Germany	?S
20	*Phanaeus labreae* (Pierce, 1946)	U Pleistocene	0.068–0.004 (pit: 0.03–0.009)	O’Keefe et al. (2009)	La Brea tar pits, U.S.A.	S
21	*Phanaeus violetae* Zunino, 2013	U Pleistocene	0.035–0.010	Clapperton (1993)	Cangahua Formation, Ecuador	S
	**Doubtful fossil Scarabaeinae**					
1	*Ateuchites grandis* Meunier, 1898	U Oligocene	28.1–23.03	Butzmann & Fischer (2013)	Armissan, Aude, France	DS,L
2	*Ateuchus ebenium* (Horn, 1876)	M Pleistocene	0.75–0.5	Daeschler, Spamer & Parris (1993) and Bechtel et al. (2005)	Port Kennedy caves, U.S.A.	DS
3	*Copris subterraneus* Heer, 1862	M Miocene	14–13.5	Berger et al. (2005) and Kälin et al. (2001)	Öhningen, Germany	DS
4	*Cretonitis copripes* Nikolajev, 2007	L Cretaceous	139.8–113.0	Zherikhin et al. (1999)	Baysa (Baissa), Russia	DS
5	*Gymnopleurus deperditus* Heer, 1862	M Miocene	14–13.5	Berger et al. (2005) and Kälin et al. (2001)	Öhningen, Germany	DS
6	*Gymnopleurus eocaenicus* Meunier, 1921	M Eocene	47	Franzen (2005) and Merz & Renne (2005)	Messel, Germany	DS, L
7	*Oniticellus amplicollis* Heer, 1862	M Miocene	14–13.5	Berger et al. (2005) and Kälin et al. (2001)	Öhningen, Germany	DS
8	*Onitis magus* Heyden, 1862	U Oligocene	25	Koenigswald et al. (1996)	Rott, Germany	DS
9	*Onthophagus luteus* Oustalet, 1874	U Oligocene	25–23	Nury & Schreiber (1997)	Aix en Provence, France	DS
10	*Onthophagus spitsbergeniensis* Krell, 2010	M–U Palaeocene	61.6–56	Manum & Throndsen (1986) and Wappler et al. (2013)	Spitsbergen, Norway	DS
11	*Onthophagus urusheeri* Krell, 2000a	M Miocene	14–13.5	Berger et al. (2005) and Kälin et al. (2001)	Öhningen, Germany	DS
12	*Phanaeus antiquus* Horn, 1876	M Pleistocene	0.75–0.5	Daeschler, Spamer & Parris (1993) and Bechtel et al. (2005)	Port Kennedy caves, U.S.A.	DS
13	*Prionocephale deplanate* Lin, 1980	U Cretaceous	91–83.6	Chen & Chang (1994), Lin (1994) and Chen (2003)	Zheijang, China	DS
14	*Scelocopris enertheus* Zhang, 1989	L–M Miocene	16.4–14.2	Yang et al. (2007)	Shanwang, China	DS

#### Character matrix

We scored the examined scarabaeine fossils (*Lobateuchus parisii, Canthochilum philipsivieorum* and *C. alleni*) into the character matrix previously developed for extant Scarabaeinae (Tarasov & Génier, 2015). The remaining described fossils, including the ones from La Brea, are too incomplete (preserving information allowing to score just one or two characters at best) to integrate into existing character matrices, preventing us from determining their phylogenetic affinities. The character matrix was constructed using Mesquite ver. 3.03 (Maddison & Maddison, 2015) and includes 114 taxa and 205 characters; it can be downloaded as Supplemental Information 1 or from MorphoBank (http://www.morphobank.org project 2184). Since we did not add any new characters to the matrix of Tarasov & Génier (2015) and did not modify its general structure, the character report for the present matrix is the same as in Tarasov & Génier (2015) and we refer the reader to that paper for details.

#### Phylogenetic analyses

For the phylogenetic inference we applied Bayesian inference in addition to the parsimony approach that is traditionally used in morphology. Bayesian analysis samples topologies from their posterior probability (PP), thus explicitly assigning a probability score to every sampled split of lineages. Comparison of probability scores between alternative splits allows straightforward evaluation of alternative positions for taxa. Parsimony analysis may also infer numerous trees, but in contrast to Bayesian inference, the split frequency in parsimony does not bear any explicit statistical explanation. Even if numerous trees are inferred in a parsimony analysis, the alternative positions of splits are not evaluated and usually one specific position for a split tends to be shown—either by using a consensus tree or by showing a “preferred” topology. The evaluation of alternative placements using the Bayesian approach is especially interesting for fossil taxa as they usually have many missing entries in the matrix, which tends to generate numerous alternative placements.

In this paper, we ran both Bayesian and parsimony analyses with two versions of the data matrix: one including all characters and one with ambiguous characters (characters #122, 71, 73, 74, 161, 204) excluded as suggested in Tarasov & Génier (2015).

#### Parsimony (MP)

The parsimony analysis was conducted in TNT ver. 1.1 (Goloboff, Farris & Nixon, 2008) under equal weights using the following TNT settings: traditional search with 3,000 replications and up to 200 trees saved per replication, tree buffer set to store 1 M trees, TBR, trees automatically condensed after search, the default collapsing rule was used.

To assign support values onto branches of the consensus trees, we calculated Bremer support values (BSV) by searching suboptimal trees up to 10 steps longer than the shortest one using TBR swapping on the shortest trees.

#### Bayesian Inference (BI)

Autapomorphic characters of terminal taxa were excluded and the data matrix was not partitioned, as suggested in Tarasov & Génier (2015). We ran MrBayes (Ronquist et al., 2012b) using the default priors, *Mk* + *Γ* model and the following options: *ngen* = *10 M*, *samplefreq* = *1 K*, *nruns* = *2*, *nchains* = *4*, and *temp* = *0.1*.

To summarize sampled trees and frequencies after burnin we used the *sumt* command with *minpartfreq* = *0.01* in order to include rare splits in the posterior sample. We used the resulting *.tstat* and *.parts* files to extract and analyse information about splits and their probabilities. The alternative splits for fossil species are shown in Fig. 2. Due to illustrational constraints confining visualization to 2D space, we demonstrate only those alternatives that are not nested within hierarchically higher splits. We call such splits elementary. We choose this illustration approach over other methods (e.g., networks or density trees) as it improves readability of the results in the present case and provides a good summary of the alternative relationships.

#### Nomenclatural acts

The electronic version of record of this article in Portable Document Format (PDF) will represent a published work according to the International Code of Zoological Nomenclature (ICZN), and hence the new names contained in the electronic version are effectively published under that Code from the electronic edition alone. This published work and the nomenclatural acts it contains have been registered in ZooBank, the Official Registry of Zoological Nomenclature. The ZooBank LSIDs (Life Science Identifiers) can be resolved and the associated information viewed through any standard web browser by appending the LSID to the prefix http://zoobank.org/. The LSID for this publication is: urn:lsid:zoobank.org:pub:D2719A53-A7C1-4D9D-BE94-FED25F17DC7C. The work is archived in PubMed Central and CLOCKSS.

## Results

### Systematic paleontology

Family: Scarabaeidae Latreille, 1802
Subfamily: Scarabaeinae Latreille, 1802
Genus: *Canthochilum* Chapin, 1934
urn:lsid:zoobank.org:act:861178F5-8EC7-4DEB-B84E-D99824185A33.

The genus *Canthochilum* is a Greater Antillean endemic comprising 23 extant species (Philips & Ivie, 2008) in addition to the two extinct species described here. The fossils of the two specimens described here were initially found and subsequently purchased on eBay. Because eBay is known to be extensively flooded with fake amber insects, we needed to critically examine the authenticity of our amber and specimens prior to species description. The proof of authenticity of amber requires sophisticated analytical techniques (Eriksson & Poinar, 2015). We ran simple tests which are usually sufficient to confirm authenticity as suggested in Eriksson & Poinar (2015). The results that alcohol drops did not affect amber pieces while burning of amber released a smell of pine resin align well with properties of authentic amber. Additionally, we consider the described *Canthochilum* fossils to be authentic because *Canthochilum* is a relatively rare, non-dung-dwelling taxon that is difficult to collect. Most specimens are caught with flight intercept traps (Philips & Ivie, 2008). Furthermore, its distribution is restricted to the Greater Antilles. Thus, it would require an entomology expert to fake those pieces of amber, which does not seem plausible. Moreover, one fossil, *C. philipsivieorum*, is very distinct from all known extant species of *Canthochilum*, thus reinforcing its fossil nature.

**Figure 1:**
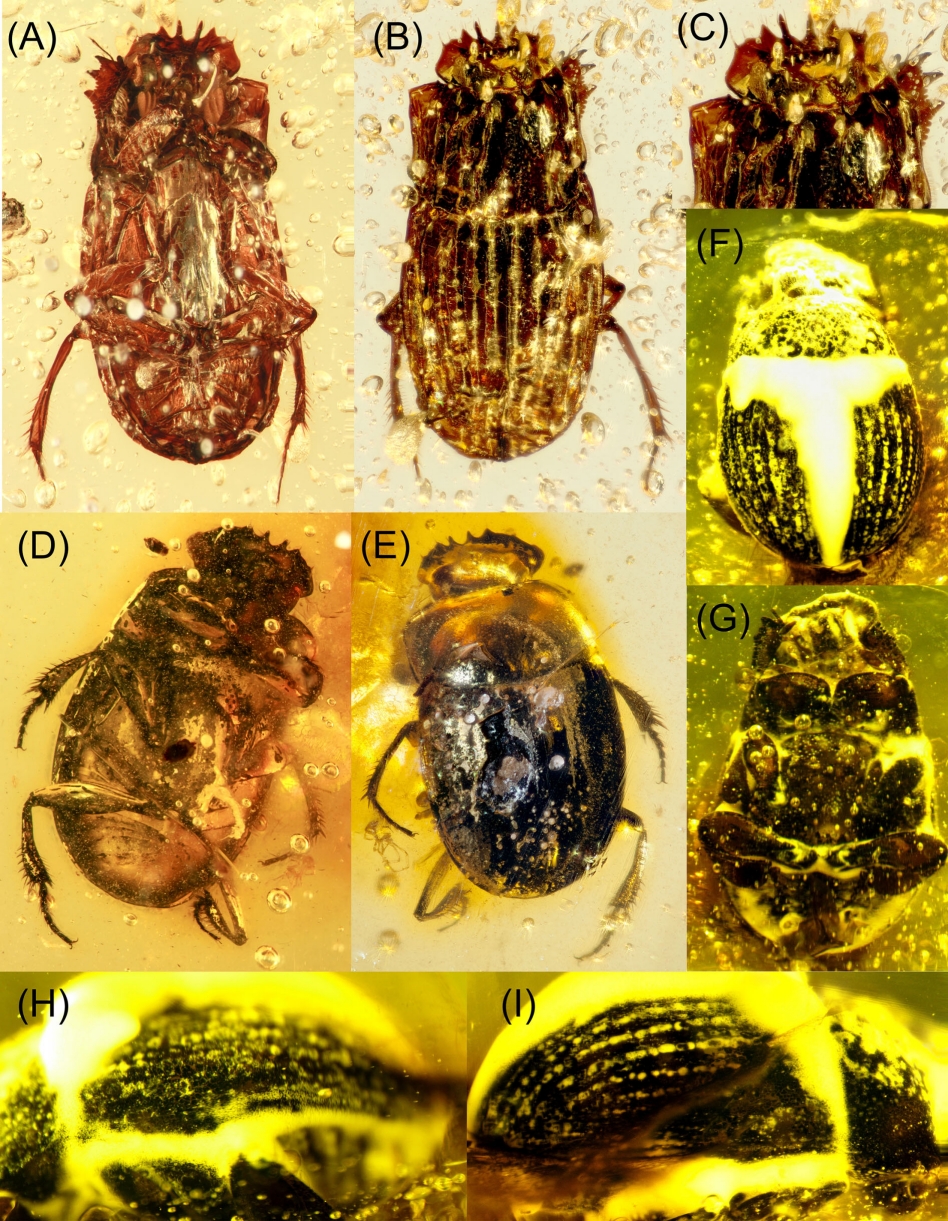
Fossil Scarabaeinae from amber. (A–C) *Canthochilum philipsivieorum* sp.n.; (D–E) *Canthochilum alleni* sp.n.; (F–I) *Lobateuchus parisii*; (A), (D) and (G) ventral view; (B), (E) and (F) dorsal view; (C) head; (H) left lateral view of elytra; (I) right lateral view of elytra.

*Canthochilum alleni* Tarasov & Vaz-de-Mello **sp.n.**
urn:lsid:zoobank.org:act:37C08BBB-FACE-42A0-BA80-DD2105347781 (Figs. 1D and 1E).

**Material examined.** Holotype, ♂, amber fossil, Dominican Republic, no additional data (CEMT).

**Description.** Body oblong, black, without visible colour differences between legs and remaining body surface.

Head: dorsal surface without any visible sculpturing, completely smooth (no visible punctures); clypeus with 4 teeth; mesal pair of teeth triangular, tips blunt; lateral pair of teeth obtuse, significantly wider than mesal teeth; clypeal margin angulate between lateral tooth and genal junction, not forming a tooth; clypeo-genal projection present and easily discernible; dorsal ocular area quite large, eyes separated by a distance equal to four times their transverse width.

Pronotum with surface covered by regular simple punctures, separated by about 3–7 puncture diameters; surface between punctures smooth.

Elytra with 8 striae, with finely shagreened surface and without visible punctures; 1st, 2nd and 8th elytral striae distinct, striae 3–7 not distinctly discernible; all elytral intervals flat except 1st which is very slightly convex; elytra with slight lateral carina that internally adjoins 7th stria; 7th stria appears to be formed by large punctures becoming distinct posteriorly.

Proleg with trochanterofemoral pit; protibia with 3 teeth, tips of basal and medial teeth separated by 1.5 times the distance between tips of apical and medial teeth; protibial spur spatulate and blunt, reaching third tarsomere.

Metacoxal lateral margin not expanded.
Metatibia only slightly curved.
Meso-metasternal suture obtusely angled.

Pygidial margins not discernable in examined specimen; pygidial disc moderately convex, shagreened, covered with scattered simple punctures.

Length: 3.9 mm.

**Observational note.** The specimen is a male, indicated by the modified shape of the protibial apical spur.

**Diagnosis.** This species is most similar to other *Canthochilum* species occurring in Hispaniola. It can be separated from other *Canthochilum* by the following combination of characters: (1) elytra with slightly expressed lateral carina, (2) body uniformly black, (3) clypeal margin with 4 teeth, (4) clypeo-genal projections present, (5) clypeal margin angulate between lateral tooth and genal junction, (6) eyes separated by a distance equal to four times their transverse width, (7) proleg with trochanterofemoral pit.

In Philips & Ivie’s (2008) key to *Canthochilum* from Hispaniola, this new species falls into couplet 6 and can be distinguished from the other two species in this couplet (*C. magnum* Philips & Ivie, 2008 and *C. darlingtoni* Matthews, 1969) by the clypeal margin being angulate between the lateral tooth and genal junction.

**Etymology.** This species is named after Albert Allen (Boise, Idaho, USA), who very kindly sent it to FVM as a donation.

**Locality and age.** The precise locality cannot be determined as the specimen was bought on eBay. The age of Dominican amber has been somewhat controversial. While older ages had been suggested previously (34–38 Ma: Dilcher, Herendeen & Hueber, 1992; 23–30 Ma: Grimaldi, 1995), Iturralde-Vinent (2001) restricted the age of fossiliferous Dominican amber to 15–20 Ma, most likely close to 16 Ma (Miocene). This restriction seems to have been largely accepted (Grimaldi & Engel, 2005; Penney, 2010).

*Canthochilum philipsivieorum* Tarasov & Vaz-de-Mello **sp.n.**
urn:lsid:zoobank.org:act:CC0D3832-40E3-43EE-8FF7-E310E0811AA6 (Figs. 1A–1C).

**Material examined.** *Holotype* (*sex unidentified*), amber fossil, Dominican Republic, no additional data (CEMT).

**Description.** Body elongate with subparallel sides, brown with metallic sheen, colouration not differ between legs and remaining body except antennal clubs yellow.

Head: dorsal surface punctate, punctures simple, visible only on frons between eyes; clypeal margin with 4 teeth, mesal pair of teeth long, acute, subparallel, lateral pair of teeth triangular, acute and almost twice as short as mesal one; clypeal margin slightly angulate between lateral tooth and genal junction, without forming a tooth; clypeo-genal projection forms small acute tooth; eyes separated by a distance equal to two times their transverse width. It is noteworthy that the frons is elevated over the rest of the head and that the clypeo-frontal area forms a distinct carina; however, we tend to treat these two structures as likely to be artefacts of deformation.

Pronotum quadrate, anterior angles straight, surface covered by simple punctures, posterior fourth of midline with trace of depression.

Elytral striae finely margined by double carina; elytral intervals slightly convex (not clearly visible due to deformations); elytra with 8 striae, external edge of 7th stria strongly carinate.

Meso-metasternal suture obtusely angled; metasternum grooved posteriorly.

Proleg with trochanterofemoral pit; protibia with 3 teeth, tips of basal and medial teeth separated by 1.5 times the distance between tips of apical and medial teeth; lateral outer margin of tibia denticulate; protibial spur spatulate with acute tip with a downward hook; spur reaches third tarsomere.

Metacoxal lateral margin not expanded.
Metatibia strongly curved in the middle.
Length: 3.8 mm.

**Observational note.** The sex of this specimen cannot be identified. Although the apical spur of its protibia is spatulate and hooked downward, which might be considered a male trait, the degree of spur modification is not sufficiently extreme to rule out the female sex.

The holotype described here was subjected to taphonomic deformations that caused numerous longitudinal carinae across the entire body by squeezing the surface of the exoskeleton. These deformations are sometimes difficult to distinguish from the actual beetle morphology. In order to filter the deformations out, we used the bilateral symmetry of beetles and treated structures as artefacts if they were not symmetrical. However, in some cases the unequivocal identification of symmetry was doubtful, as mentioned in the description.

Despite the presence of strong elytral deformations that complicate observations, we assume that this species has a typical *Canthochilum* elytral morphology. Elytra in *Canthochilum* have a total of 8 striae. In some species, the external edge of the 7th stria adjoins either the longitudinal elytral carina or its trace or just the abruptly angulate edge. The presence of a carina in this species is supported by strong, bilaterally symmetrical (although deformed) elevations next to the 7th stria, while the presence of an 8th stria is suggested by a lateral row of setose punctures visible apically (the characteristic feature of many *Canthochilum* species). We conclude that this species has elytra with 8 striae and a strong carina that internally adjoins the 7th stria.

**Diagnosis.** This species can be unequivocally distinguished from all other *Canthochilum* species by its distinct elongate body shape and by the clypeal teeth forming a long, mesal pair of teeth and a shorter pair of lateral triangular teeth.

**Etymology.** This name derives from the combination of the last names of our colleagues Keith Philips and Michael Ivie, who worked extensively on the taxonomy of the genus *Canthochilum*.

**Locality and age.** See above under *Canthochilum alleni*.

### Phylogenetic analyses

Parsimony analysis of the full dataset yielded 66,400 trees of length 563, while analysis with ambiguous characters excluded yielded 29,400 of length 472. The convergence between runs in both Bayesian analyses was achieved after ∼2M generations and by the end of the runs the standard deviation of split frequencies was far below the acceptable limit of 0.01.

**Figure 2:**
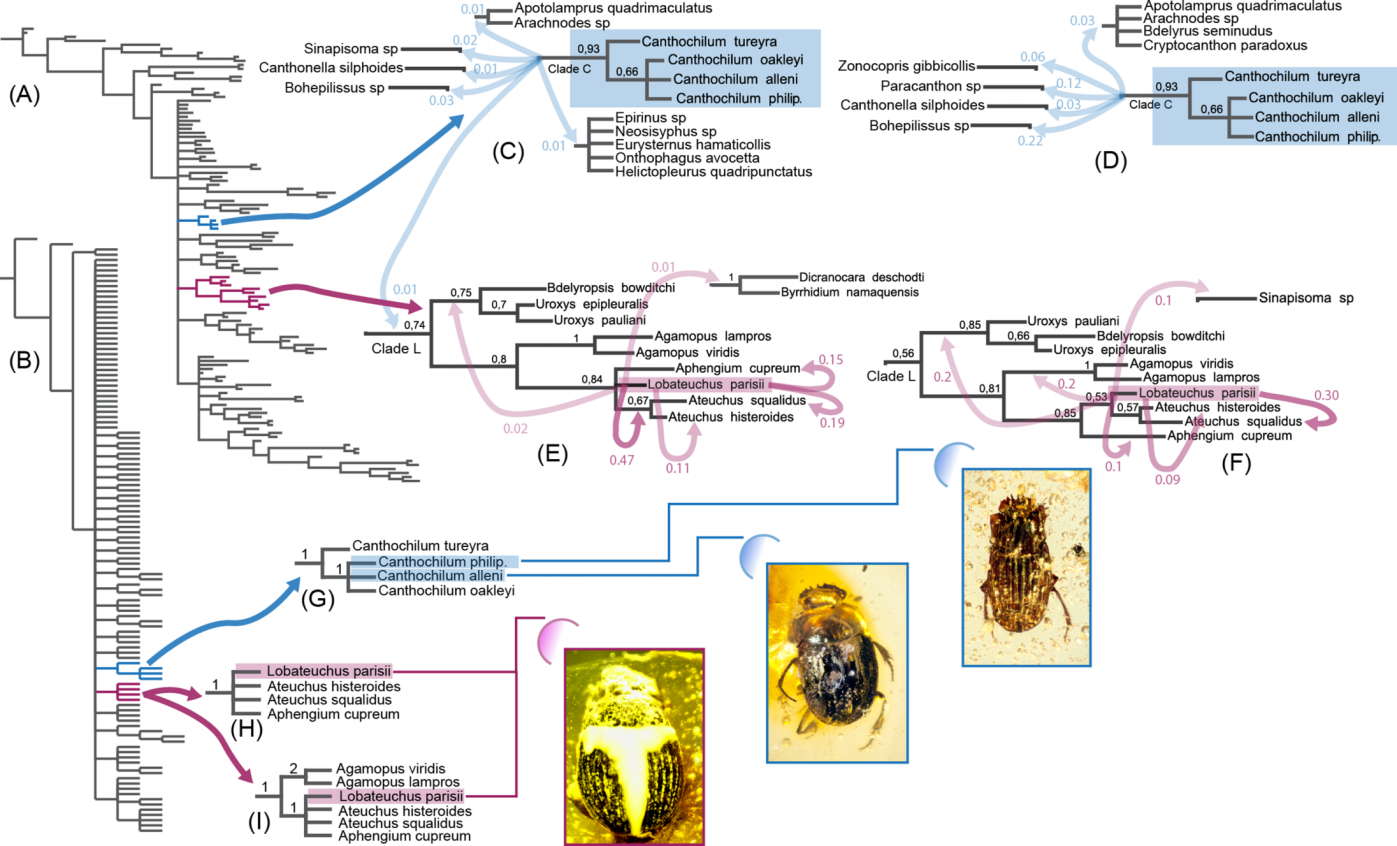
Morphology-based Bayesian and parsimony analyses of Scarabaeinae showing positions of investigated fossil species, which are illustrated in the low left corner. *Canthochilum philip.* is an abbreviation for *Canthochilum philipsivieorum.* (A) Bayesian 50% majority consensus tree from the analysis with all characters included, tips labels are removed; (B) Parsimony tree from the analysis with all characters included, tips labels are removed; (C) *Canthochilum* clade from Bayesian analysis with all characters included and the alternative elementary splits it forms; (D) *Canthochilum* clade from Bayesian analysis with ambiguous characters excluded and the alternative elementary splits it forms; (E) *Lobateuchus* and allies clade from Bayesian analysis with all characters included and the alternative elementary splits it forms; (F) *Lobateuchus* and allies clade from Bayesian analysis with ambiguous characters excluded and the alternative elementary splits it forms; (G) *Canthochilum* from two parsimony analyses (with and without ambiguous characters) and BSV; (H) *Lobateuchus* and allies clade from parsimony analysis with all characters included (BSV are shown); (I) *Lobateuchus* and allies clade from parsimony analysis without ambiguous characters included (BSV are shown).

Bayesian and parsimony analyses recovered similar phylogenetic placements of the fossil species. *Lobateuchus parisii* emerged within a clade formed by *Ateuchus* + *Aphengium* in both the analyses including all characters (BSV = 1, PP = 0.84) and the analyses with ambiguous characters excluded (BSV = 1, PP = 0.81) (Figs. 2E, 2F, 2H and 2I). The two fossil species of *Canthochilum* were recovered within a clade comprising two extant *Canthochilum* species, with the same support values in the analyses with and without ambiguous characters (BSV = 1, PP = 0.93) (Figs. 2C, 2D and 2G).

Alternative positions of fossil species in Bayesian analyses generally have significantly lower support than those revealed by the 50% majority consensus tree, nevertheless they are worth mentioning since they directly reflect the distribution of topological variability of the results. It is noteworthy that inclusion/exclusion of characters did not significantly change the results and mainly affected splits with low PP.

In the Bayesian analyses, *Lobateuchus parisii* is nested within the larger clade L (Figs. 2E and 2F), which also includes *Ateuchus, Aphengium, Agamopus, Uroxys and Bdelyropsis*. In turn the clade L forms a polytomy with many other dung beetle lineages and usually branches off early in the sampled trees. Most frequently, *Lobateuchus* forms a sister group with two *Ateuchus* species in both the analysis without ambiguous characters (PP = 0.53) and the analysis with ambiguous characters (PP = 0.47). Some splits support its sister position with taxa of the clade L, among which the most notable are: *Ateuchus squalidus* (PP_with ambiguous_ = 0.19, PP_without ambiguous_ = 0.30), Aphengium (PP_with ambiguous_ = 0.15, PP_without ambiguous_ = 0.1) and *Agamopus* (PP_without ambiguous_ = 0.2). The remaining alternative splits illustrated in Figs. 2E and 2F have very low posterior probabilities.

The clade formed by *Canthochilum* species (including the two fossils) came up monophyletic with moderate posterior support (PP = 0.93). Interestingly the sample of the analysed splits does not contain any alternatives that support a non-monophyletic pattern for this clade due to the fact that such alternative splits have PP < 0.01 and therefore are not included in the summary statistics (*sumt minpartfreq* = *0.01*) and are not discussed here. Since *Canthochilum* shows stable monophyly in our analyses, we focus our discussion on the alternative positions for this genus. The clade forming *Canthochilum* (clade C) makes up a polytomy with other scarabaeine clades, indicating the high uncertainty of the relationships between *Canthochilum* and other lineages. Among elementary splits the highest PP is found between *Canthochilum* and *Bohepilissus* (PP_without ambiguous_ = 0.22) as well as *Canthochilum* and *Paracanthon* (PP_without ambiguous_ = 0.12). All the remaining elementary splits are rare with posterior probabilities < 0.1. In addition to *Canthochilum* itself, these splits are formed by such taxa as *Sinapisoma*, *Canthonella*, *Zonocopris*, *Arachnodes,* and *Apotolamprus*.

## Discussion

### Phylogenetic position of fossil Scarabaeinae from amber

The amber fossils *Lobateuchus* and *Canthochilum* are the best preserved scarabaeine fossils. Moreover, *Lobateuchus* represents the oldest reliable dung beetle fossil (see next section), which makes their phylogenetic assessment valuable for uncovering dung beetle evolutionary history.

The phylogenetic position of *Lobateuchus* (Figs. 1F–1I) in the same clade with *Ateuchus* and *Aphengium* is stable and supported in both the Bayesian and parsimony analyses. The authors who originally described *Lobateuchus* (Montreuil, Génier & Nel, 2010) probably noticed the body shape similarity between *Ateuchus* and *Lobateuchus*, thus deriving the name of the latter from the former and indirectly pointing out their similarity. However, in the discussion of the taxonomic position they suggest that *Lobateuchus* seems closely related to *Haroldius*. Our current morphological phylogeny lacks any representatives of *Haroldius,* thus precluding the test of this hypothesis.

However, the relatively high PP value supporting a nested position of *Lobateuchus* in the clade containing *Ateuchus* and *Aphengium* as inferred in present analyses must also be taken with caution because of data deficiency. Many important parts of *Lobateuchus* are covered with a layer of white impurities and hence hidden from direct observation. The piece of amber embedding the fossil is cracked and since it contains the holotype of *Lobateuchus,* it is preserved in a tightly closed glass capsule filled with Canada balsam for permanent storage. Capsule destruction and amber cleaning are needed to make the fossil specimen available for micro-CT investigation. These procedures along with potentially destructive power of x-rays significantly increase the risk of destroying the holotype specimen. Due to this reasons and MNHN policy the holotype was not available for tomography (decision of the curator of insect fossils at MNHN). It is unknown how much data would be available with tomography investigation (we doubt it would be much) but the investigation with stereomicroscope resulted in missing data for this taxon in the data matrix (72%, or 147 characters), which can bias the results of the analyses. To demonstrate this, let us evaluate the obtained results from the point of their character support. Clade *Ateuchus* + *Aphengium* is supported by one unique and three homoplasious synapomorphies (Tarasov & Génier, 2015). Two of the homoplasious synapomorphies are also preserved in *Lobateuchus*: epipleuron slightly protruded downward submedially and proleg lacking trochanterofemoral pit. In the analyses, these two synapomorphies appear to link *Lobateuchus* with *Ateuchus* + *Aphengium,* while the alternative placements are poorly supported due to extensive missing data. Thus, the clade formed by *Lobateuchus, Ateuchus* and *Aphengium* appears to be determined only by the characters that remain preserved in *Lobateuchus.* Although many phylogenetically important characters of internal body structures and male genitalia cannot be observed in *Lobateuchus*, the present evidence for the position of *Lobateuchus* is the best we could obtain. However, the limitations associated with the inference of the systematic placement of *Lobateuchus* must be kept in mind.

Unlike *Lobateuchus*, the placement of the two fossil *Canthochilum* species within the genus is well supported by both parsimony and Bayesian analyses, despite the extent of missing data (∼60% for each). The *Canthochilum* lineage is defined by two unique synapomorphies: the extremely reduced parameres and the highly modified shape of the aedeagal sclerite (Tarasov & Génier, 2015). None of these characters is observable in the fossil specimens, but in addition to these two synapomorphies *Canthochilum* species also share a unique set of diagnostic characters: (1) elytron with 8 distinctly visible striae; and (2) pro-, meso-, and metatarsus and apex of meso- and metatibia distinctly setose. In addition to these characters, some *Canthochilum* have the internal margin of the lateral elytral carina adjoining the 7th stria. The set of these three diagnostic characters is present in both described fossils, thus providing their well-corroborated position in *Canthochilum* by the phylogenetic analyses.

Notably, the body shape of the fossil *C. alleni* looks exactly like that of many extant *Canthochilum* species (e.g., C. *magnum* Philips & Ivie, 2008 and *C. darlingtoni* Matthews, 1969; see Philips & Ivie, 2008). Given an age for Dominican amber of 16 Ma, such close similarity points to a slow rate of morphological evolution in at least some *Canthochilum* lineages. The second fossil *C. philipsivieorum* differs from all other *Canthochilum* by its elongated body shape, but despite numerous deformations of its exoskeleton, all diagnostic *Canthochilum* characters are present in this species, which strongly supports its placement in *Canthochilum*.

The phylogenetic position of *Canthochilum* within Scarabaeinae, however, remains unresolved. *Canthochilum* forms a polytomy at the base of the scarabaeine tree in both parsimony and Bayesian consensus trees (Figs. 2A and 2B). This polytomy is caused by the high uncertainty in sister-group relationships between *Canthochilum* and other main scarabaeine lineages. The alternative sister relationships for *Canthochilum* inferred by the Bayesian analyses (shown in Figs. 2C and 2D) suggest that this genus tends to cluster with genera such as *Canthonella*, *Bohepilissus, Paracanthon* and *Zonocopris*. This is consistent with the implied weight parsimony tree inferred by Tarasov & Génier (2015). *Canthochilum* species have not yet been included in any published molecular phylogeny, which makes such molecular analyses highly welcome in order to elucidate the placement of *Canthochilum.*

We believe that our character matrix provides a solid base for future studies of the phylogenetic relationships of extinct dung beetles. The updated catalogue of fossil Scarabaeinae and the detailed assessment of their placement should also be a useful tool for future studies of scarabaeine evolution and systematics.

### Review of Scarabaeinae fossils

#### Doubtful vs. Reliable Fossils

The fossil record of Scarabaeinae is poor, most likely due to their largely dung-associated lifestyle as living below the surface in terrestrial environments makes fossilization far less probable. Currently, the fossil record of Scarabaeinae comprises 35 described fossils (including the ones described in this study; see Catalogue section and Table 1). We also have evidence of “deltochiline-like” fossil(s) in Baltic amber which was unavailable for this study as we are in the process of locating the specimen. In the catalogue section below, we assess each fossil species based on its original description and illustrations and attempt to conclude whether its position in Scarabaeinae and its generic placement can be considered doubtful or reliable.

Out of the 35 described fossil species, 14 cannot be confidently considered as scarabaeines due to the lack of any preserved character(s) that would unequivocally support their placement in Scarabaeinae; these taxa must therefore be treated as doubtful scarabaeines or even placed outside Scarabaeinae. Here we prefer to treat them as doubtful dung beetles whose placement in Scarabaeinae must be considered highly questionable. Contemporary revisions of old fossil descriptions usually tend to reduce the number of species described in a given taxon. For example, a recent revision of hydrophilid beetles described by Heer (1862) from the Öhningen locality in Germany, known for its rich insect deposits (Selmeier, 1990), revealed numerous inaccurate family and genus attributions (Fikácek & Schmied, 2013). Heer (1862) described four scarabaeine species from Öhningen that we consider doubtful: (*Copris subterraneus* Heer, 1862; *Gymnopleurus deperditus* Heer, 1862; *Oniticellus amplicollis* Heer, 1862; and *Onthophagus urusheeri* Krell, 2000a).

We treat the remaining 21 fossil species as reliable Scarabaeinae (Table 1) and use them to reexamine the scarabaeine fossil record. Most of them appear to have unambiguous generic affiliations; they belong to extant genera such as *Onthophagus, Eodrepanus, Phanaeus, Heliocopris, Copris* and *Canthochilum*, except for the oldest reliable fossil, belonging to the extinct genus *Lobateuchus*. A few fossils (*Anachalcos mfwangani* Paulian, 1976; *Copris leakeyorum* Paulian, 1976; *Copris druidum* Heer, 1862; *Gymnopleurus rotundatus* Heer, 1862 and *Metacatharsius rusingae* Paulian, 1976) from the localities in Lake Victoria (Paulian, 1976) and Öhningen, Germany (Heer, 1862) can be reliably considered as scarabaeines, but their generic placement will require a separate investigation of the specimens. Since this is beyond the scope of the present study, we adopt the generic placements used in their original descriptions or subsequent revisions.

Five extinct dung beetle genera have been described. We place four of them, namely, *Ateuchites, Cretonitis, Prionocephale* and *Scelocopris,* in the category of doubtful Scarabaeinae.

#### Distribution of fossils

**Figure 3:**
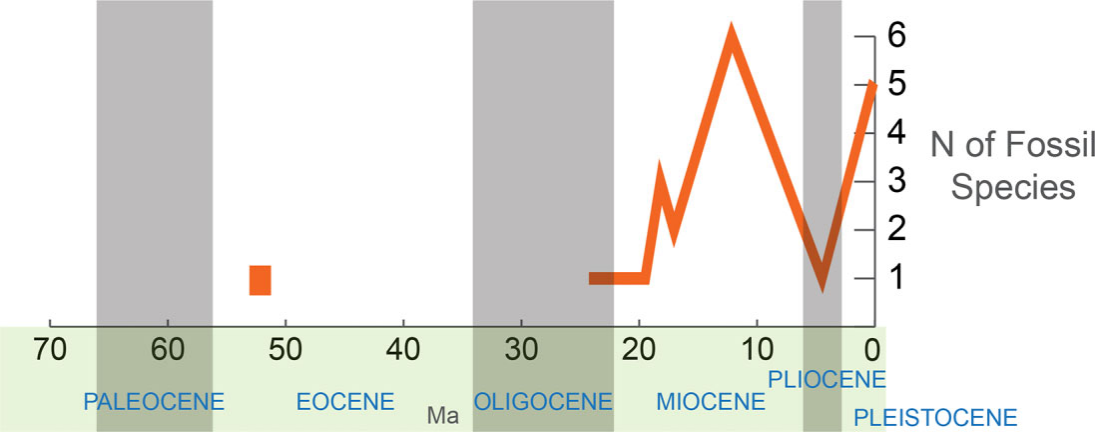
Sampled diversity of Scarabaeinae fossils over geological time.

The highest concentration of reliably identified scarabaeine fossils (Fig. 3) is observed in the Miocene (14 species; incl. 1 Mio–Pliocene), followed by the Pleistocene (5 fossil species). One fossil is known from the Oligocene (Rott, Gemany), and another (*Lobateuchus*), representing the oldest reliable dung beetle, dates back to the Eocene (53 Ma). The fossils are not uniformly distributed over the phylogenetic tree (e.g., that in Tarasov & Génier, 2015) and some taxa, namely Onthophagini + Oniticellini, *Copris*, and *Phanaeus,* have the largest number of fossil species (2–7 each). Fossil taxa tend to be concentrated in the large clade that includes *Metacatharsius, Gymnopleurus, Anachalcos, Heliocopris* and Onthophagini + Oniticellini likely due to the higher probability of fossilization in species-rich groups.

The fossils described from the late Oligocene onwards can all be placed within extant genera, with many of them resembling extant species. This indicates that the main extant phylogenetic lineages of Scarabaeinae had at least evolved by the late Oligocene–mid Miocene (28–12 Ma).

Biogeographically, the majority of the fossils (16 species) are known from the Old World. However, the generally poor record of dung beetle fossils and the ambiguous taxonomic placement for several of them do not allow us to draw any biogeographic conclusions.

### Scarabaeinae age based on fossil data

The oldest known fossils described as Scarabaeinae are *Cretonitis copripes* and *Prionocephale deplanate,* from the Lower and Upper Cretaceous, respectively. Fossilized dung beetle brood balls of the ichnogenus *Coprinisphaera* were recorded by Hasiotis (1999) and Hasiotis (2004) from the Upper Jurassic Morisson Formation in the Rocky Mountain Region of the U.S.A., but Bromley et al. (2007) questioned the interpretation of those fossils. A *Coprinisphaera* from the Lower Jurassic of Sołtyków, Poland, is neither described nor figured (Pieńkowski, 2004). Another *Coprinisphaera* specimen was recovered from the Upper Cretaceous Adamantina Formation in Brazil by Carvalho, Gracioso & Fernandes (2009). In addition, an ichnofossil representing a dinosaur coprolite with associated dung-filled tunnels similar to those made by dung beetles is known from the Cretaceous (Chin & Gill, 1996). These few inconclusive specimens are the only fossil record of putative dung beetles from the Mesozoic. As reliable identification and inference of the taxonomic position of *Cretonitis* and *Prionocephale* is not currently possible (see Catalogue section), the attribution of these Mesozoic fossils to Scarabaeinae is doubtful. The fossil brood balls need closer examination, but misinterpretation of spherical structures due to their scarcity of characters is easily possible. This means that currently “there are no reliable fossils or fossilized evidence which would support a Mesozoic origin for [scarabaeine] dung beetles” (Tarasov & Génier, 2015). The investigation of all described fossils conducted in this paper reveals that the oldest reliable scarabaeine fossil is *Lobateuchus parisii,* known from the Eocene (53 Ma). We suggest using this Eocene fossil in all relevant assessments of the minimum age for Scarabaeinae based on fossil data. Nevertheless, this Eocene fossil does not rule out the earlier origin of dung beetles. A recent comprehensive molecular phylogeny of Coleoptera (McKenna et al., 2015) that used calibration points from other beetle fossils dates the origin of Scarabaeinae at Upper Cretaceous which seems quite plausible. Contrary to that, the dating of dung beetle tree using published mutation rates (not fossil-based calibration points) yields relatively younger ages referring to Late Paleocene–Eocene: ∼56.5 Ma (Sole & Scholtz, 2010) and ∼45.3 Ma (Mlambo, Sole & Scholtz, 2015).

## Catalogue of Fossil Scarabaeinae

This is an updated version of the catalogue of fossil Scarabaeinae, largely based on Krell (2000a) and Krell (2007). The fossil species are placed under two sections in alphabetic order. The first section lists species that we can confidently assign to Scarabaeinae, while the second lists those with doubtful assignment (see also Table 1). For every species we provide our justification explaining why it can or cannot be considered a scarabaeine, and we also express our concern if the current generic position of the species in Scarabaeinae needs to be separately investigated. Such species with doubtful generic position are marked with “?” before their genus names. The plausibility assessment for all species (except *Lobateuchus* and *Canthochilum*) is based on the literature data and illustrations provided in the original descriptions, and, for some of Heer’s specimens, by photographs of the specimens.

### Fossils confidently assigned to Scarabaeinae

#### ?Anachalcos mfwangani Paulian, 1976

*Anachalcos mfwangani* Paulian, 1976: 1 (Miocene, Lake Victoria, Kenya).–Krell, 2000a: 879; Krell, 2007: 19.

**Note.** The body shape of this three-dimensional fossil suggests its placement in Scarabaeinae. However, the generic position inside Scarabaeinae is doubtful–the poor preservation of this fossil, as noted in the original description, obscures the necessary diagnostic characters.

#### Canthochilum alleni sp.n.

*Canthochilum alleni* **sp.n.** (L Miocene, Burdigalian, Dominican amber, Dominican Republic).

**Note.** The species is described herein; see Systematic Paleontology section.

#### Canthochilum philipsivieorum sp.n.

*Canthochilum philipsivieorum* **sp.n.** (L Miocene, Burdigalian, Dominican amber, Dominican Republic).

**Note.** The species is described herein; see Systematic Paleontology section.

#### Copris (Copris) kartlinus Kabakov, 1988

*Copris (Copris) kartlinus* Kabakov, 1988: 110 (U Miocene–L Pliocene, Kisatibi/Goderdzi Formation, Georgia).–Krell, 2000a: 879; Krell, 2007: 20; Krell & Schawaller, 2011: 540.

**Note.** The well-preserved imprint of this fossil unequivocally supports its placement in the genus *Copris*.

#### ?Copris druidum Heer, 1862

*Copris druidum* Heer, 1862: 73, pl. 6 (M Miocene, Öhningen, Kesselstein, Germany).–Heer, 1865: 378f; Heer, 1883: 404f; Scudder, 1891: 500; Handlirsch, 1906–1908: 837; Krell, 2000a: 879; Krell, 2007: 20.

**Note.** The original description with body shape illustration support the placement of this fossil in Scarabaeinae. The confirmation of the generic attribution to *Copris* would require additional investigation.

#### ?Copris leakeyorum Paulian, 1976

*Copris leakeyorum* Paulian, 1976: 1 (Miocene, Lake Victoria, Kenya).–Krell, 2000a: 879; Krell, 2007: 20.

**Note.** The body shape of this three-dimensional fossil suggests its placement in Scarabaeinae. However, the generic position inside Scarabaeinae is doubtful–the poor preservation of this fossil, as noted in the original description, obscures the necessary diagnostic characters.

#### Copris pristinus Pierce, 1946

*Copris pristinus* Pierce, 1946: 124; (U Pleistocene, Rancho La Brea [tar pits], Los Angeles, U.S.A.).–Halffter, 1959: 176; Matthews, 1961: 35, 67, 69; Matthews & Halffter, 1968: 160 (*rembuchei*-group); Sphon, 1973: 52; Miller, Gordon & Howden, 1981: 626; Stock & Harris, 1992: 70, 84; Miller, 1997: 188; Krell, 2000a: 879; Ashworth, 2001: 159; Ashworth, 2003; Morón, 2003: 12; Morón, 2004: 166; Krell, 2006: 132; Krell, 2007: 20; Elias, 2010: 5, 230.

**Note.** The well-illustrated original description (Pierce, 1946) and subsequent investigations (Matthews & Halffter, 1968; Miller, Gordon & Howden, 1981) strongly support the validity of this species and its placement in *Copris*.

#### *Eodrepanus coopei* Barbero, Palestrini & Roggero, 2009

*Eodrepanus coopei* Barbero, Palestrini & Roggero, 2009: 1853 (U Pleistocene, Eemian, Trafalgar Square, London, UK).

**Note.** The well-preserved remains of elytra and pronotum support placement of this fossil in *Eodrepanus* (see Barbero, Palestrini & Roggero, 2009). Similar fragments possibly belonging to the same species were previously found by Gao et al. (2000) in Eemian deposits of the River Great Ouse in Cambridgeshire, UK.

#### *?Gymnopleurus rotundatus* Heer, 1862

*Gymnopleurus rotundatus* Heer, 1862: 73, pl. 6 (M Miocene, Öhningen, Kesselstein, Germany). Heer, 1865: 378f; Heer, 1883: 404f; Scudder, 1891: 527; Handlirsch, 1906–1908: 837; Meunier, 1921: 11; Krell, 2000a: 879.

**Figure 4:**
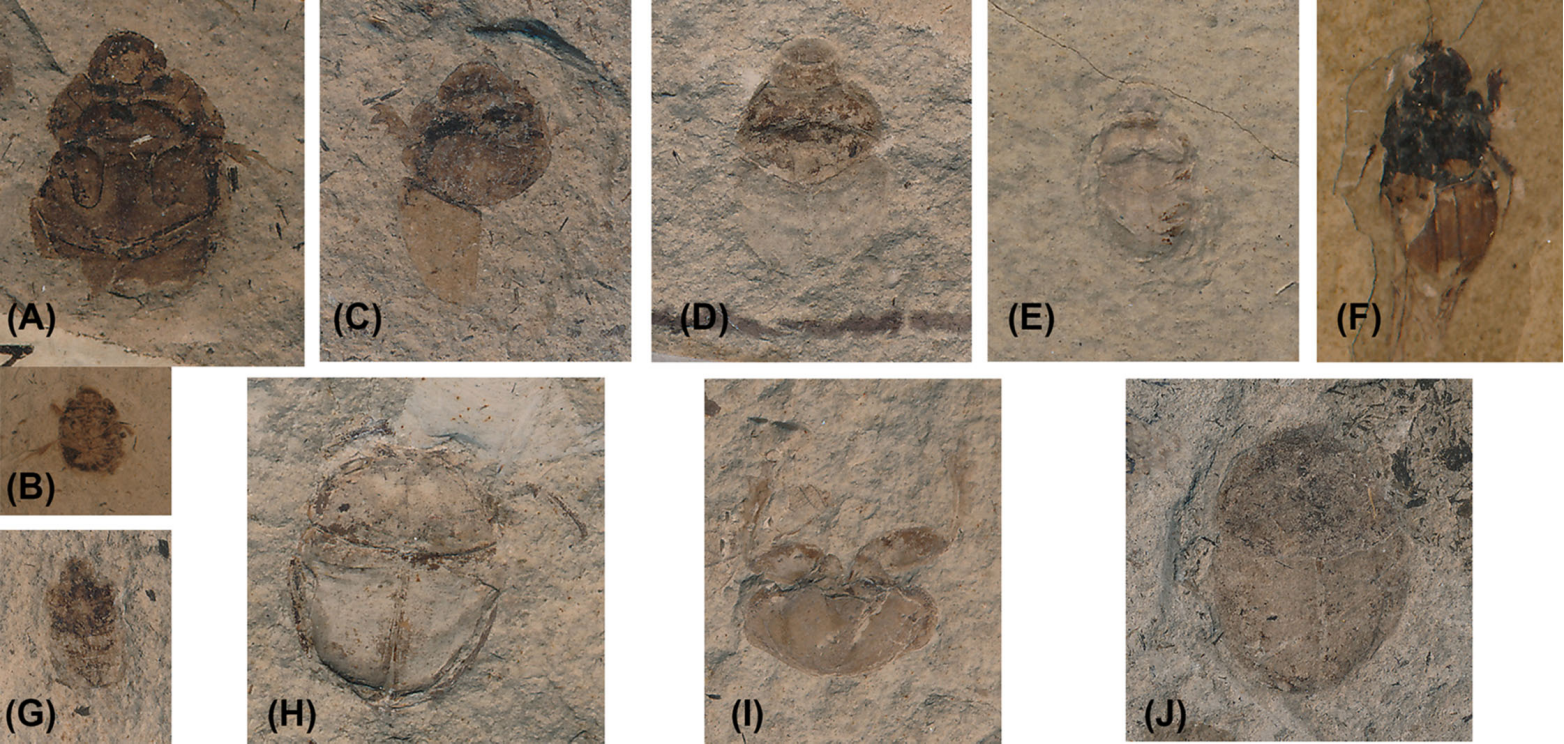
Some fossil Scarabaeinae described by O. Heer. All fossils are described from the Late Miocene (14–13.5 Ma) locality of Öhningen in Germany. (A) and (B) Syntypes of *Onthophagus crassus*; (C) and (D) Syntypes of *Onthophagus prodromus*; (E) Holotype of *Onthophagus ovatulus*; (F) Type of *Onthophagus bisontinus*; (G) Holotype of *Oniticellus amplicollis*; (H) Holotype of *Gymnopleurus sisyphus*; (I) Holotype of *Gymnopleurus deperditus*; (J) Putative type of *Gymnopleurus rotundatus*.

**Note.** The species is described from two specimens. The body shape and teeth of the anterior edge of the protibia of one specimen is mentioned in the original description (Heer, 1862). The photograph of the other specimen, which putatively belongs to the type series, shows the body shape (Fig. 4J). Those characters support the placement of this species within the Scarabaeinae. However, the poor preservation and lack of diagnostic characters at the generic level raise doubts about the generic placement of this species, and investigation of the type series is be needed to properly assess its generic placement and conspecificity.

#### *Gymnopleurus sisyphus* Heer, 1847

*Gymnopleurus sisyphus* Heer, 1847: 64, pl. 7 (M Miocene, Öhningen, Germany).–Bronn, 1849: 625; Stitzenberger, 1851: 100; Giebel, 1852: 653; Giebel, 1856: 38; Heer, 1862: 72; Scudder, 1891: 527; Handlirsch, 1906–1908: 839; Meunier, 1921: 11f; Krell, 2000a: 879; Krell, 2007: 20.

**Note.** The body shape and lateral elytral notch (Fig. 4H) strongly support the placement of this fossil in the genus *Gymnopleurus*.

#### *Heliocopris antiquus* Fujiyama, 1968

*Heliocopris antiquus* Fujiyama, 1968: 203 (L Miocene, Yanagida Formation, Noto, Japan).–Harusawa, 1994: 23; Krell, 2000a: 879; Krell, 2007: 21.

**Note.** The description of this species is based on an incomplete fossil specimen consisting of elytra and part of the hind legs. The thorough original description and good illustrations provide solid evidence that this fossil is a true *Heliocopris*. This placement is supported by large elytron size, shape of tibiae and tarsi, and elytra with at least 7 striae (6 of which can be directly observed in the fossil, while traces of the 7th stria are observable on the internal margin of the lateral elytral carina).

#### Genus *LOBATEUCHUS* Montreuil, Génier & Nel, 2010

*Lobateuchus* Montreuil, Génier & Nel, 2010: 165 (type species by original designation: *Lobateuchus parisii* Montreuil, Génier & Nel, 2010).

#### *Lobateuchus parisii* Montreuil, Génier & Nel, 2010

*Lobateuchus parisii* Montreuil, Génier & Nel, 2010: 165 (L Eocene, Ypresian, Oise amber, France).–Nel & Brasero, 2010: 147.

**Note.** A detailed overview of this fossil is provided in previous sections.

#### *?Metacatharsius rusingae* Paulian, 1976

*Metacatharsius rusingae* Paulian, 1976: 2 (Miocene, Lake Victoria, Kenya).–Krell, 2000a: 879; Krell, 2007: 21.

**Note.** The body shape of this three-dimensional fossil suggests its placement within Scarabaeinae. However, the generic position within Scarabaeinae is doubtful–the poor preservation of this fossil, as noted in the original description, obscures the necessary diagnostic characters.

#### *Onthophagus bisontinus* Heer, 1862

*Onthophagus bisontinus* Heer, 1862: 76, pl. 76 (M Miocene, Öhningen, “Insektenschicht des unteren Bruches,” Germany).–Heer, 1865: 379; Heer, 1883: 405; Scudder, 1891: 559; Handlirsch, 1906–1908: 837; Krell, 2000a: 880; Krell, 2007: 21.

**Note.** The morphology of this fossil resembles typical representatives of the genus *Onthophagus* (Fig. 4F), where it seems most closely related to the members of the subgenus *Proagoderus*.

#### *?Onthophagus crassus* Heer, 1862

*Onthophagus crassus* Heer, 1862: 75, pl. 6 (M Miocene, Öhningen, Kesselstein, Germany).–Heer, 1865: 379; Heer, 1883: 405; Oustalet, 1874: 196; Scudder, 1891: 559; Handlirsch, 1906–1908: 837; Krell, 2000a: 880; Krell, 2007: 21.

**Note.** The type series comprises two specimens, both of which can be reliably assigned to Onthophagini (Figs. 4A and 4B) but which may represent different species. Presently, the tribe Onthophagini comprises a few dozen genus-group taxa with obscure taxonomic limits and rank (genus vs. subgenus). The diagnoses for many of them are poorly defined and need to be phylogenetically revised. At this moment, the generic placement of fossil Onthophagini is impossible. Following the original description by tentatively placing this species in *Onthophagus,* the type genus of the tribe, seems at present to be the most conservative course of action.

#### *Onthophagus everestae* Pierce, 1946

*Onthophagus everestae* Pierce, 1946: 131 (U Pleistocene, Rancho La Brea [tar pits], Los Angeles, U.S.A.).–Sphon, 1973: 52; Miller, Gordon & Howden, 1981: 627f; Wilson, 1986: 101; Stock & Harris, 1992: 70, 84; Miller, 1997: 187f; Krell, 2000a: 880; Ashworth, 2001: 159; Ashworth, 2003; Krell, 2006: 132; Krell, 2007: 21; Elias, 2010: 5, 230.

**Note.** The remains of this fossil are well-preserved; its original description (Pierce, 1946) and subsequent investigation (Miller, Gordon & Howden, 1981) support the validity of this species and its placement in *Onthophagus*.

#### *?Onthophagus ovatulus* Heer, 1847

*Onthophagus ovatulus* Heer, 1847: 64, pl. 7 (M Miocene, Öhningen, Germany).–Bronn, 1849: 624; Giebel, 1852: 653; Giebel, 1856: 39; Heer, 1865: 379; Heer, 1883: 405; Scudder, 1891: 559; Handlirsch, 1906–1908: 837; Krell, 2000a: 880; Krell, 2007: 22.

**Note.** Based on the photo of the holotype (Fig. 4E), this species should likely be assigned to Onthophagini. However, an examination of the fossil specimen is required to clarify its taxonomic position within the tribe. The problems associated with placement of this fossil species in *Onthophagus* are the same as those discussed for *O. crassus*.

#### *?Onthophagus prodromus* Heer, 1862

*Onthophagus prodromus* Heer, 1862: 75, pl. 6 (M Miocene, Öhningen, Kesselstein, Germany).–Heer, 1865: 378f; Heer, 1883: 404f; Scudder, 1881–1885: 795; Oustalet, 1874: 196; Scudder, 1891: 559; Handlirsch, 1906–1908: 837; Krell, 2000a: 880; Krell, 2007: 22.

**Note.** The type series comprises a few specimens, two of which are illustrated in the original description and were also seen by us in photographs (Figs. 4C and 4D). These can be reliably assigned to Onthophagini but do not seem conspecific. One of the fossils (Fig. 4C) resembles species of *Onthophagus* subgenus *Digitonthophagus* (specifically *O. gazella* or *O. bonasus*) The generic placement of fossil Onthophagini is discussed in *O. crassus.*

#### *?Onthophagus statzi* Krell, 1990

*Onthophagus muelleri* Statz, 1952: 8 (U Oligocene, Chattian, Rott, Germany), preoccupied by *Onthophagus muelleri* Novak, 1921: 99).

*Onthophagus mulleri.*–Sphon, 1973: 52.

*Onthophagus statzi* Krell, 1990: 187 (replacement name).–Krell, 2000a: 880; Krell, 2007: 22.

**Note.** Based on the illustration of the body shape (Statz, 1952), this species should likely be assigned to Onthophagini. However, examination of the fossil specimen is required to clarify its taxonomic position within the tribe. The problems associated with placement of this fossil species in *Onthophagus* are the same as those discussed for *O. bisontinus*.

#### *Phanaeus labreae* (Pierce, 1946) Miller, Gordon & Howden, 1981

*Palaeocopris labreae* Pierce, 1946: 130 (U Pleistocene, Rancho La Brea [tar pits], Los Angeles, U.S.A.).–Matthews, 1961: 35 (“appears to be a composite of two genera”); Sphon, 1973: 52; Stock & Harris, 1992: 84; Wilson, 1986: 101; Krell, 2007: 22.

*Paleocopris labreae.*–Halffter, 1959: 176.

*Phanaeus labreae*.–Miller, Gordon & Howden, 1981: 627; Krell, 2000a: 880; Krell, 2006: 132; Price, 2009: 148.

**Note.** The well-preserved fossil can unequivocally be placed in Scarabaeinae. The original description (Pierce, 1946) assigned this species to a separate genus *Palaeocopris* Pierce, 1946. Subsequent investigation (Miller, Gordon & Howden, 1981) supported the validity of this species but revealed the synonymy of *Palaeocopris* with *Phanaeus*.

#### *Phanaeus violetae* Zunino, 2013

*Phanaeus violetae* Zunino, 2013: 221 (U Pleistocene, Cangahua Formation, Quito, Ecuador).

**Note.** This fossil is known by a well-preserved complete head, which strongly supports its placement in *Phanaeus*.

### Doubtful fossil Scarabaeinae

#### Genus *ATEUCHITES* Meunier, 1921

*Ateuchites* Meunier, 1898: 114 (type species by monotypy: *Ateuchites grandis* Meunier, 1898).–Handlirsch, 1925: 246; Théodoridès, 1952: 34; Balthasar, 1963: 79; Iablokoff-Khnzorian, 1977: 137; Carpenter, 1992: 300; Krell, 2000a: 879; Paetel, 2001: 234; Krell, 2007: 19; Scholtz, 2009b: 32, 34.

#### *Ateuchites grandis* Meunier, 1898

*Ateuchites grandis* Meunier, 1898: 114 (U Oligocene, Chattian, Armissan, Aude, France).–Handlirsch, 1906–1908: 836; Carpenter, 1992: 300; Krell, 2000a: 879; Krell, 2007: 19.

**Note.** According to Martin Nose, curator at Bayerische Staatssammlung für Paläontologie und Geologie, München (where this species would likely be deposited), this fossil seems to be lost. The insufficient original description does not contain any characters which could shed light on the taxonomic position of this species or even support its placement within the superfamily Scarabaeoidea. We have to conclude that the taxonomic placement of this fossil remains a mystery and cannot support or refute its membership in Scarabaeinae.

#### *?Ateuchus ebenius* (Horn, 1876) Daeschler, Spamer & Parris, 1993

*Choeridium ebenium* Horn, 1876: 245 (M Pleistocene, Irvingtonian, Port Kennedy caves, Pennsylvania, U.S.A.).–Scudder, 1890: 490, pl. 1; Wickham, 1920: 358; Théodoridès, 1952: 36; Krell, 2000a: 879; Krell, 2006: 132; Krell, 2007: 20.

*Choeridium*? *ebenium* Horn, 1876.–Lesley, 1889: xiii; Scudder, 1891: 490; Scudder, 1900: 104; Handlirsch, 1906–1908: 1126.

*Choeridium*? *[= Ateuchus] ebenium* Horn, 1876.–Daeschler, Spamer & Parris, 1993: 31.

Since *Choeridium* Le Peletier de Sait-Fargeau & Audinet-Serville, 1828 was synonymized with *Ateuchus* Weber, 1801 by Chapin (1946); Horn’s species has to be included in the latter, even if its generic assignment is doubtful.

**Note.** The limited original description (Horn, 1876) lacks any reasonable character that could unequivocally support the placement of this fossil in Scarabaeinae. Additionally, Horn (1876) seemed to hesitate assigning this species to Scarabaeinae, which likely indicates that poor preservation obscured the necessary diagnostic characters. Therefore, we doubt the assignment of this fossil to Scarabaeinae, but first-hand examination of the specimen is required to reach a substantiated conclusion.

#### *?Copris subterraneus* Heer, 1862

*Copris subterranea* Heer, 1862: 74, pl. 6 (M Miocene, Öhningen, Kesselstein, Germany).–Heer, 1865: 379; Heer, 1883: 405; Scudder, 1891: 501; Handlirsch, 1906–1908: 837; Krell, 2000a: 879; Krell, 2007: 20.

**Note.** The original description indicated that the species is known by the imprint of elytra, which precludes any conclusion on its taxonomic affiliation and, at the same time, challenges its position in Scarabaeinae since elytra lack any clear diagnostic characters.

#### Genus *CRETONITIS* Nikolajev, 2007

*Cretonitis* Nikolajev, 2007: 131 (type species by original designation: *Cretonitis copripes* Nikolajev, 2007).

#### *Cretonitis copripes* Nikolajev, 2007

*Cretonitis copripes* Nikolajev, 2007: 132, 221 (L Cretaceous, Valanginian–Aptian, Zaza Formation, Baysa, Russia).–Tarasov & Génier, 2015: 32.

*Cretonitis ikhbogdensis* Nikolajev, 2007.–Nomen nudum, lapsus calami, Nikolajev, 2007: 215.

**Note.** As was pointed out earlier (Tarasov & Génier, 2015) the original description is based solely on the incomplete impression of one middle leg. While the leg shape of this fossil resembles that of Tribe Onitini, this similarity must be taken with caution as Scarabaeinae lack any unique diagnostic characters on the middle leg. Since the assignment of this fossil to Scarabaeinae is lacking solid evidence, we tentatively consider it as doubtful Scarabaeinae.

#### *?Gymnopleurus deperditus* Heer, 1862

*Gymnopleurus deperditus* Heer, 1862: 73, pl. 6 (M Miocene, Öhningen, Kesselstein, Germany).–Handlirsch, 1906–1908: 836; Meunier, 1921: 11; Krell, 2000a: 879; Krell, 2007: 20.

**Note.** The fossil consists of a prothorax with preserved forelegs (Fig. 4I). Their shapes likely support the placement of this fossil in the superfamily Scarabaeoidea. The incompleteness of the fossil prevents the precise inference of its taxonomic affiliation.

#### *?Gymnopleurus eocaenicus* Meunier, 1921

*Gymnopleurus eocaenicus* Meunier, 1921: 12, pl. 3 (M Eocene, Lutetian, Messel, Germany).–Koenigswald, 1987: 140; Krell, 2000a: 879; Paetel, 2001: 234; Wedmann, 2005: 106; Krell, 2007: 20.

*Gymnopleurus eocenicus.*–Théodoridès, 1952: 46.

**Note.** The preservation of this fossil was poor. The specimen could not be traced in 1999 in the Meunier collection at the Hessisches Landesmuseum Darmstadt and is likely to be lost. The shape of the fossil illustrated in the original description does not resemble a representative of Scarabaeinae (or even of Coleoptera) at all.

#### *?Oniticellus amplicollis* Heer, 1862

*Oniticellus amplicollis* Heer, 1862: 76, pl. 6 (M Miocene, Öhningen, Kesselstein, Germany).–Heer, 1865: 378f; Heer, 1883: 404f; Scudder, 1891: 558; Handlirsch, 1906–1908: 837; Krell, 2000a: 879, Krell, 2000b: 177; Krell, 2007: 21.

**Note.** The original description and illustration (Fig. 4G) do not provide any characters that can justify the placement of this fossil in Scarabaeinae. The elongated pronotum that is almost as long as the elytra and the big scutellum indicate that this species might be closely related to the *Aphodius* lineages (Aphodiinae) sharing a large scutellum.

#### *?Onitis magus* Heyden, 1862

*Onitis magus* Heyden, 1862: 65, pl. 10 (U Oligocene, Chattian, Rott, Germany).–Krantz, 1867: 315; Scudder, 1891: 558; Handlirsch, 1906–1908: 837; Krell, 2000a: 879; Krell, 2007: 21.

*Onitis magnus*.–Statz, 1952: 2.

**Note.** According to Janssens in Balthasar (1963: 79) this fossil rather represents a species of *Zonitis* Fabricius, 1775 from a different beetle family, Meloidae. Heyden may have confused two similar names, and erroneously assigned this fossil to the scarabaeine genus *Onitis*. The original description illustrates a body shape atypical for Scarabaeiodea. The slender tibiae without denticles also support this conclusion.

#### *?Onthophagus luteus* Oustalet, 1874

*Onthophagus luteus* Oustalet, 1874: 194, pl. 2 (U Oligocene, U Chattian, Aix-en-Provence, France).–Goss, 1878: 339; Scudder, 1891: 559; Handlirsch, 1906–1908: 837; Théobald, 1937: tabl. 11; Théodoridès, 1952: 46; Krell, 2000a: 880.

**Note.** The original description accompanied by an illustration lacks any reasonable characters supporting placement of this species in Onthophagini or, more generally, in Scarabaeoidea. The examination of fossil specimens is needed to properly assess its position. Given the lack of character support, this fossil should likely be removed from Scarabaeinae.

#### *?Onthophagus spitsbergeniensis* Krell, 2010

*Elytridium rugulosum* Heer, 1870: 78, pl. 16 (M–U Palaeocene, Spitzbergen, Norway) (suppressed, see International Commission on Zoological Nomenclature, 2011).

*Onthophagus rugulosus* (nec Harold in Heyden, Harold & Kraatz (1886: 78)).–Birket-Smith, 1977: 25; Krell, 2007: 46 (doubtful); Krell, 2010: 29 (Onthophagini).

*Onthophagus spitsbergeniensis* Krell, 2010: 29 (replacement name).–International Commission on Zoological Nomenclature, 2011: 218.

**Note.** The fossil is known from one elytron that indeed resembles those in the scarabaeine genus *Onthophagus* or in the tribe Onthophagini in general (Birket-Smith, 1977; Krell, 2010). However, the presence of only one elytron puts this fossil in a data deficient category where a reliable inference of the taxonomic position is impossible. Therefore, we mark this fossil as doubtful Scarabaeinae.

#### *?Onthophagus urusheeri* Krell, 2000a

*Onthophagus urus* Heer, 1847: 62, pl. 2 (M Miocene, Öhningen, Germany).–Bronn, 1849: 624; Stitzenberger, 1851: 100; Giebel, 1852: 653; Giebel, 1856: 39; Heer, 1862: 76; Heer, 1865: 379; Heer, 1883: 405; Scudder, 1891: 559; Handlirsch, 1906–1908: 837; preoccupied by *Onthophagus urus* Ménétries, 1832: 175.

*Onthophagus urusheeri* Krell, 2000a: 880 (replacement name).–Krell, 2007: 22.

**Note.** Unfortunately, the type of this specimen was not located in Heer’s collection in ETH. The illustration provided in the original description is of poor quality (Heer, 1847), which does not allow us assessing its taxonomic position.

#### *?Phanaeus antiquus* Horn, 1876

*Phanaeus antiquus* Horn, 1876: 245 (M Pleistocene, Irvingtonian, Port Kennedy caves, Pennsylvania, U.S.A.).–Goss, 1878: 340; Scudder, 1890: 489, pl. 1; Scudder, 1891: 565; Scudder, 1900: 104; Handlirsch, 1906–1908: 1126; Wickham, 1920: 358; Théodoridès, 1952: 36; Krell, 2000a: 880; Krell, 2006: 132; Krell, 2007: 22; Price, 2009: 148.

*Phanaeus antiquum* Horn, 1876.–Daeschler, Spamer & Parris, 1993: 31.

**Note.** Same as in *Ateuchus ebenius.*

#### Genus *PRIONOCEPHALE* Lin, 1980

*Prionocephale* Lin, 1980: 230 (type species by original designation: *Prionocephale deplanate* Lin, 1980).–Krell, 2000a: 880; Paetel, 2001: 234; Krell, 2007: 22; Nikolajev, 2007: 130, 215.

#### *Prionocephale deplanate* Lin, 1980

*Prionocephale deplanate* Lin, 1980: 230 (U Cretaceous, U Turonian–Santonian, Lanxi Formation, Zheijang, China).–Lin, 1994: 314; Krell, 2000a: 880; Krell, 2006: 131, 133; Krell, 2007: 22; Nikolajev, 2007: 214; Scholtz, 2009b: 30f, 34; Jingjing et al. 2010: 210; Philips, 2011: 33; Tarasov & Génier, 2015: 32.

*Prionocephale deplanae.*–Lin, 1983: 394.

**Note.** While the fossil is poorly preserved, some characters, such as the Scarabaeini-like head with a strongly denticulated clypeus, the strongly denticulated front legs, and the Onthophagini-/Scarabaeini- or *Circellium*-like body shape suggest the possibility of this species being a member of the Scarabaeinae. However, apart from body shape and general adaptation for digging, this fossil lacks any diagnostic characters that could unambiguously support its placement in Scarabaeinae or even the superfamily Scarabaeoidea. Although examination of the fossil specimen is needed to clarify its affinities, due to insufficient preservation we doubt that it will reveal any new critical characters. Until a better-preserved specimen can be confidently assigned to Scarabaeinae, we consider this fossil as doubtful Scarabaeinae and suggest caution when using it for assessing the age of dung beetles.

#### Genus *SCELOCOPRIS* Zhang, 1989

*Scelocopris* Zhang, 1989: 150, 425 (type species by original designation: *Scelocopris enertheus* Zhang, 1989).–Krell, 2000a: 880; Paetel, 2001: 234; Krell, 2007: 22; Scholtz, 2009a: 32.

#### *Scelocopris enertheus* Zhang, 1989

*Scelocopris enertheus* Zhang, 1989: 151, 425 (Miocene, Shanwang Formation, China).–Krell, 2000a: 880; Krell, 2007: 23.

**Note.** The placement of this fossil in Scarabaeinae is doubtful. The body shape provided in the original illustrations differs from the general scarabaeine form. The original description does point out that the hind tibia has only one apical spur, which is a characteristic of Scarabaeinae; however, the visibility of only one apical spur can be the result of incomplete preservation. Examination of the fossil specimen is needed to confirm its placement in Scarabaeinae and infer its taxonomic position.

## Supplemental Information

10.7717/peerj.1988/supp-1 Supplemental Information 1 Matrix S1. The character matrix used in phylogenetic analyses.

## Additional Information and Declarations

### Data Deposition

The following information was supplied regarding data availability: Character matrix is deposited on MorphoBank (http://www.morphobank.org project 2184).

### New Species Registration

The following information was supplied regarding the registration of a newly described species: Genus LSID: *Canthochilum* urn:lsid:zoobank.org:act:861178F5-8EC7-4DEB-B84E-D99824185A33; *Canthochilum alleni* LSID urn:lsid:zoobank.org:act:37C08BBB-FACE-42A0-BA80-DD2105347781; *Canthochilum philipsivieorum* LSID urn:lsid:zoobank.org:act:CC0D3832-40E3-43EE-8FF7-E310E0811AA6; Publication LSID: urn:lsid:zoobank.org:pub:D2719A53-A7C1-4D9D-BE94-FED25F17DC7C.
